# Association of Plant-Based Dietary Patterns with Activities of Daily Living Disability in Older Adults Based on a 10-Year Cohort Study

**DOI:** 10.3390/nu16234011

**Published:** 2024-11-23

**Authors:** Dahuan Cai, Yanxin Zeng, Xiao Liang, Anchao Song, Mengliang Ye

**Affiliations:** School of Public Health, Chongqing Medical University, No. 1 Yixueyuan Road, Yuzhong District, Chongqing 400016, China; 2023121636@stu.cqmu.edu.cn (D.C.); m18323173972@163.com (Y.Z.); liangxiao8061217@163.com (X.L.); soongac@cqmu.edu.cn (A.S.)

**Keywords:** plant-based dietary patterns, Activities of Daily Living Disability, older adults, cohort study, Cox proportional hazards model

## Abstract

Background: In the context of population aging, activities of daily living (ADL) disability has brought great challenges to the health of the elderly. The relationship between plant-based dietary patterns and the health of the elderly has been widely discussed. However, few studies have explored the correlation between plant-based dietary patterns and ADL disability in older adults. Methods: We included 2004 participants from the Chinese Longitudinal Health Longevity Survey (CLHLS). The Simplified Food Frequency Questionnaire was used to measure Plant-Based Diet Index (PDI), healthy Plant-Based Diet Index (hPDI), and unhealthy Plant-Based Diet Index (uPDI). A Cox proportional hazards model was used to assess associations between plant-based dietary patterns and ADL disability in older adults and to explore age differences in these associations. Results: uPDI and hPDI showed nonlinear associations with ADL disability. Following covariate adjustments, older adults in the highest tertile of the hPDI have a lower risk of ADL disability than those in the lowest tertile (HR = 0.61). Older adults in the highest tertile of the uPDI have a higher risk of ADL disability than those in the lowest tertile (HR = 1.33). Subgroup analyses showed that hPDI was more protective against ADL disability in those under 80 years of age, whereas uPDI was only significantly associated with an increased risk of ADL disability in those under 80 years of age. Conclusions: Increasing the intake of healthy plant-based diets and reducing the intake of unhealthy plant-based and animal-based diets can help prevent and improve ADL disability in the elderly.

## 1. Introduction

The World Health Organization defines disability as an umbrella term for impairment or limitation of activity [1]. The ability to perform basic activities of daily living (ADL) is an important measure of disability [2]. According to the World Health Organization, more than 46% of older adults aged 60 years and above suffer from a disability [3]. In recent years, with the aging of China’s population, the prevalence of ADL disability among older adults aged 60 years and above in China has reached 23.8% [4]. The prevalence of ADL disability has not only caused serious impacts on patients and their families but also poses a great challenge to socioeconomic development and the public health system [5,6]. Therefore, it is crucial to explore the risk factors for disability in people’s ability to perform daily activities and to develop appropriate prevention strategies to improve their quality of life in later life and public health [7,8].

Previous studies have shown that the causes of disability in older persons include cognitive decline, heavy physical labor, poor clinical care, and poor dietary habits [7,9,10]. Dietary patterns play a pivotal role in disability prevention and health promotion in older adults [11,12,13]. Prior research indicates that plant-based dietary patterns (such as the Mediterranean and Japanese diets), which are low in fat and calories and rich in various nutrients including antioxidants (vitamins A, C, E), B vitamins, and n-3 polyunsaturated fatty acids, may mitigate the risk of cognitive impairment and cardiovascular disease in older adults, thereby potentially reducing the risk of disability in this population [10,14,15,16,17,18]. However, not all plant-based diets contribute positively to controlling risk factors for disability in older adults. Hemler et al. reported that low-quality plant-based diets can lead to a high intake of refined carbohydrates or sugars, which in turn increases the risk of chronic diseases such as cardiovascular disease in older people and is detrimental to the prevention and control of disability [19].

Although some current studies have explored the relationship between plant-based diets and various risk factors for disability [19,20,21], the association between different quality plant-based diets and ADL disability in older adults remains unclear. This study aims to examine the link between different types of plant-based diets and ADL disability among older adults. It seeks to enrich the research in this field and proposes appropriate preventive strategies aimed at improving the quality of life for older adults.

## 2. Subjects and Methods

### 2.1. Participants

The Chinese Longitudinal Health Longevity Survey (CLHLS) is a national prospective longitudinal study organized by the Center for Healthy Ageing and Development of Peking University in China. Since the baseline survey was conducted in 1998, it has been carried out every 3–4 years, with a total of 8 surveys completed by 2018, collecting a wide range of data. More information is provided on other topics [22].

**Figure 1:**
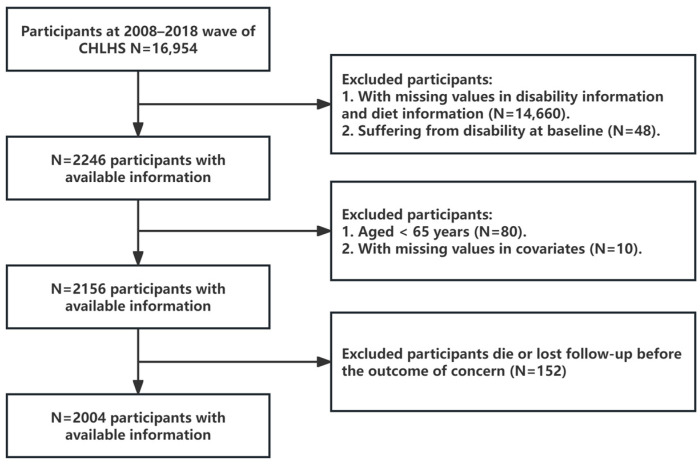
Flow chart of sample selection.

This study utilized CLHLS data from 2008 to 2018, with 16,954 participants completing the survey by 2018. This study was conducted according to the guidelines laid down in the Declaration of Helsinki and all procedures involving human patients were approved by the Biomedical Ethics Committee of Peking University (IRB00001052-13074, November 2022). Written informed consent was obtained from all patients. Initially, this study excluded participants who lacked dietary information at baseline and disability information during follow-up (14,660), as well as those with pre-existing disability at baseline (48). Subsequently, participants younger than 65 years of age were excluded (80), as were those who lacked a covariate at baseline (10). Finally, participants who were lost to follow-up or died during follow-up were also excluded (152). Ultimately, 2004 participants were enrolled in this study. Figure 1 presents a detailed flow chart of the participant inclusion and exclusion process.

### 2.2. Assessment of Disability

Disability status is assessed based on the ability to perform basic activities of daily living (ADL) [23,24]. ADL was measured through the following 6 entries: (1) bathing; (2) eating; (3) dressing; (4) indoor transfers; (5) toileting; (6) incontinence control. A participant is considered to have an ADL disability if they are unable to perform one or more of these activities without assistance from another person [2].

### 2.3. Calculation of the Plant-Based Diet Index

Dietary data were collected through a simplified food frequency questionnaire (FFQ) (as shown in Table S1), categorizing foods into two groups: plant-based foods (whole grains, nuts, tea, legumes, garlic, vegetable oils, fresh fruits, fresh vegetables, preserved vegetables, refined grains, and sugar) and animal-based foods (fish and seafood, meat, animal fat, eggs, and dairy products) [25,26]. These 16 food groups formed the basis for calculating the overall Plant-Based Diet Index (PDI), the healthy Plant-Based Diet Index (hPDI), and the unhealthy Plant-Based Diet Index (uPDI). Whole grains, fruits, vegetables, vegetable oils, legumes, garlic, nuts, and tea belong to a healthy plant-based diet, while refined grains, sugar, and preserved vegetables belong to an unhealthy plant-based diet.

Based on previous studies, we scored the PDI based on frequency of consumption [27,28]. For the PDI, the higher the frequency of consuming plant-based foods, the higher the score (with a maximum frequency of 5 and a minimum of 1). For the hPDI, the higher the frequency of consuming healthy plant-based foods, the higher the score (with a maximum frequency of 5 and a minimum of 1), and the higher the frequency of consuming unhealthy plant-based foods, the lower the score (with a maximum frequency of 1 and a minimum of 5). For uPDI, the higher the frequency of consuming unhealthy plant-based foods, the higher the score (with a maximum frequency of 5 and a minimum of 1); the higher the frequency of consuming healthy plant-based foods, the lower the score (with a maximum frequency of 1 and a minimum of 5) [29]. For animal foods, whether PDI, hPDI, or uPDI, the higher the frequency of consumption, the lower the score (with a maximum frequency of 1 and a minimum of 5). Each participant’s final PDI, hPDI, and uPDI scores were calculated from the 16 food group scores, with a theoretical range of 16 to 80 points. Participants were then categorized into three groups (T1, T2, and T3) based on their scores.

The rationale for including animal-based food in the calculation of the PDI of this study is as follows: (1) Incorporating animal-derived foods provides a more accurate reflection of the study participants’ actual dietary habits, facilitating a comprehensive assessment of their dietary patterns and allowing for a more scientifically rigorous evaluation of the health implications of a plant-based diet [20,27]. (2) Incorporating animal foods in the calculations partially addresses the limitations of previous binary classifications of dietary patterns into vegetarian and non-vegetarian categories, accounting for the role of animal-derived foods while emphasizing the significance of plant-based foods within the overall diet [30]. (3) The proportion of plant-based and animal-based diets varies across cultures and individual diets, and incorporating animal-based foods in the PDI calculation enhances its applicability across diverse cultural and personal dietary contexts.

### 2.4. Covariates

In our analyses, we adjusted for covariates that may affect ADL in older adults, including gender (male and female), age (<80 years, ≥80 years), residence (urban, town, and rural), financial status (sufficient and not sufficient), living arrangement (with a household member, solitary, and in an institution), marital status (married, separated, divorced, and widowed), drinking (yes, no), smoking status (yes, no), physical exercise (yes, no), and body mass index (underweight, normal, overweight, and obese).

### 2.5. Statistical Analysis

The study was statistically analyzed using SPSS version 27.0 and R version 4.4.1. GraphPad version 9.0 was used to visualize the results of subgroup analyses. Descriptive statistics were employed to summarize the baseline characteristics of the study population. Cox proportional hazard models were utilized to estimate hazard ratios (HRs) and their corresponding 95% confidence intervals (CI) for the association between the three plant-based diet indices and disability in activities of daily living among older adults. Restricted cubic splines were plotted to explore the potential nonlinear relationships between diet indices and ADL disability. The Akaike Information Criterion (AIC) was applied to determine the optimal number of knots for the splines [31]. The Wald test assessed the linearity of the observed relationships. Model 1 presented the univariate regression results of the plant-based diet index with ADL disability. Model 2 adjusted for additional covariates including gender, age, residence, financial status, living arrangements, marital status, smoking status, drinking status, exercise status, and body mass index (BMI), based on Model 1. Subgroup analyses based on Model 2 were performed to investigate potential differences in the association between plant-based dietary indices and ADL disability across age groups. Statistical significance was set at *p* < 0.05 for a two-sided test.

## 3. Results

### 3.1. Baseline Characteristics

A total of 2004 participants were included in this study, comprising 52.6% females and 47.4% males. The majority of the elderly participants were under 80 years of age (71.8%), resided in rural areas (67.4%), were classified as economically well-off (78.0%), and lived with their families (83.5%). By the end of the follow-up period, 28.4% of the participants had developed ADL disability. The 2018 CLHLS sample shows good internal consistency for the ADL, with a Cronbach’s alpha of 0.82. Survival curves for PDI, hPDI, and uPDI are detailed in Figures S1–S3.

In this study, significant disparities were observed in the dietary indices among older adults, specifically the PDI, hPDI, and uPDI. These disparities were found to be associated with residential area, marital status, and body mass index (BMI). Notably, the scores for PDI and hPDI were influenced by age and socioeconomic factors, including exercise habits and economic status. Furthermore, there was a significant correlation between PDI and uPDI scores with the level of ADL disability and residential conditions. Gender and smoking status were identified as additional determinants of PDI scores. All these associations were statistically significant at the *p* < 0.05 level, as detailed in Table 1 and Tables S2 and S3.

### 3.2. Association of Plant-Based Diet Index with Disability in Activities of Daily Living

**Figure 2:**
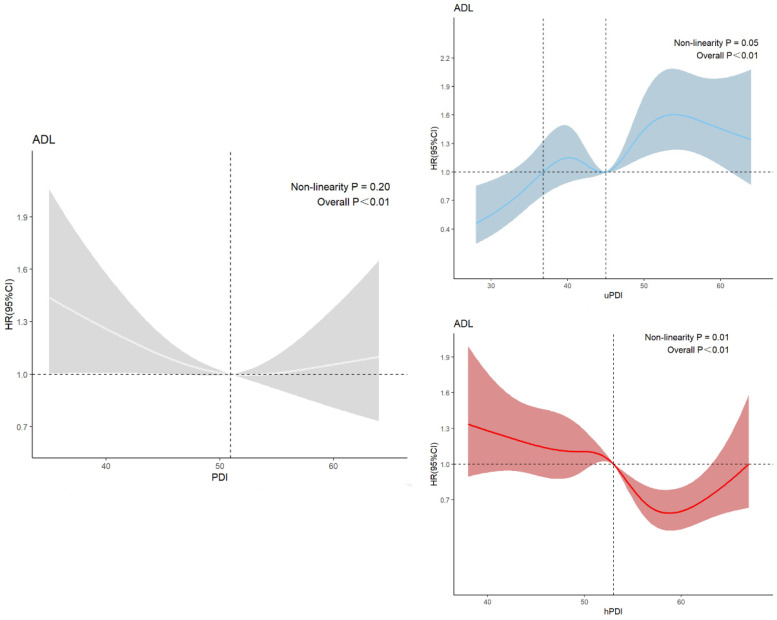
Restricted cubic curves for PDI, uPDI, hPDI. Note: ADL refers to activities of daily living; PDI refers to Plant-Based Diet Index; hPDI refers to health Plant-Based Diet Index; uPDI refers to unhealthy Plant-Based Diet Index; HR refers to hazard ratio; CI refers to confidence interval. White lines indicate associations between PDI scores and ADL disability; blue lines indicate associations between uPDI and ADL disability; and red lines indicate associations between hPDI and ADL disability. Vertical dashed lines indicate the PDI, hPDI, and uPD scores on the x-axis for HR values of 1. Shaded areas are 95% confidence intervals.

Figure 2 illustrates that the associations of the PDI, uPDI, and hPDI with ADL disability were statistically significant (*p* < 0.01). PDI demonstrated a linear relationship with ADL disability (*p* = 0.20), whereas uPDI and hPDI exhibited nonlinear relationships with ADL disability (*p* = 0.05; *p* = 0.01). In Model 1 (as shown in Table 2), compared to the first group, seniors in the PDI third group had a lower risk of developing ADL disability (HR = 0.81, *p* = 0.03). However, in Model 2, there was no statistically significant difference in the risk of ADL disability among older adults in the three PDI groups (*p* = 0.20). In Model 1, compared to the first group, seniors in the second and third hPDI group had a lower risk of developing ADL disability (HR = 0.82, *p* = 0.04; HR = 0.53, *p* < 0.01). However, in Model 2, there was no longer a statistically significant difference between the first and second hPDI groups (*p* = 0.11). In Models 1 and 2, older adults in the uPDI third group were more likely to have an ADL disability compared to older adults in the first group.

### 3.3. Age Differences in Plant-Based Diet Index and Activities of Daily Living Disability

**Figure 3:**
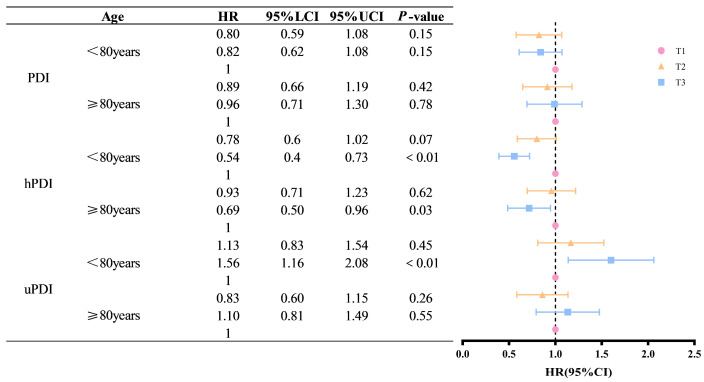
Association of age subgroups of PDI, hPDI, and uPDI with the incidence of risk of ADL disability. Note: Participants were then categorized into three groups (T1, T2, and T3) based on their scores. T1 refers to the lowest scoring of the three groups; T2 refers to the second highest scoring of the three groups; T3 refers to the highest scoring of the three groups.

To further elucidate the association of a plant-based diet with ADL disability in older adults of different ages, we conducted subgroup analyses by age (80 years and 80 years and older) (Figure 3). The results of the study showed that among older adults under 80 years of age, those in the group with the highest uPDI scores had a higher risk of ADL disability compared to those in the group with the lowest uPDI scores (HR = 1.56, *p* < 0.01). In contrast, this association was not statistically significant among the elderly aged 80 years and above (*p* = 0.55). For all older adults, those in the highest-scoring group of the hPDI were less likely to develop ADL disability than those in the lowest-scoring group (HR = 0.78, *p* < 0.01; HR = 0.69, *p* = 0.03).

## 4. Discussion

The current investigation assesses the correlation between plant-based dietary patterns and the risk of ADL disability in older adults. The study’s findings reveal a significant correlation between the overall Plant-Based Diet Index (PDI), the healthy Plant-Based Diet Index (hPDI), and the unhealthy Plant-Based Diet Index (uPDI) with the risk of ADL disability in older adults. PDI and hPDI exhibited a negative correlation with the risk of ADL disability in older adults, in contrast to the uPDI, which displayed a positive correlation with this risk. This study implies that a dietary pattern characterized by a high intake of plant-based foods and a low intake of animal-based foods may mitigate the risk of disability among older adults. Conversely, a reduction in plant-based dietary consumption in favor of an increased intake of animal-based foods is associated with an elevated risk of disability in older adults. In addition, we identified age-related disparities in the correlation between the hPDI and the uPDI with the risk of ADL disability among older adults. Elevated levels of the hPDI were found to confer a more robust protective influence on the risk of ADL disability in older adults aged less than 80, compared to their counterparts aged 80 and older. Conversely, elevated levels of the uPDI were only significantly linked to a heightened risk of ADL disability in older adults aged less than 80 years.

The current study reveals that a high hPDI is associated with a lower risk of ADL disability in older adults, findings that align with those of preceding studies. Gazerani et al. have demonstrated that chronic degenerative diseases (CDDs)—including obesity, cardiovascular disease, chronic obstructive pulmonary disease (COPD), inflammation, cognitive decline, and bone fractures—are the primary contributors to disability among older adults [32,33,34]. However, a healthy plant-based diet holds significant value in both preventing and mitigating chronic degenerative diseases [35,36,37]. Alysha et al. have reported that adherence to a healthy plant-based diet can enhance cardiovascular health, decrease the incidence of cardiovascular disease, and alleviate their detrimental effects, consequently lowering the risk of disability attributable to such conditions [25,38,39,40]. This may be because a healthy plant-based diet reduces the intake of substances such as sodium, cholesterol, and saturated fat and increases the intake of highly unsaturated fat and low saturated fat, thereby reducing the blood concentrations of total cholesterol, LDL, and non-HDL cholesterol [26,41]. Additionally, comparative analyses from prior research indicate that plant-based diets, particularly those that are healthy, are enriched with higher concentrations of antioxidants and anti-inflammatory nutrients compared to animal-based diets [42,43]. These diets are believed to moderate oxidative and inflammatory stress in the nervous system of older adults and thus ameliorate cognitive decline in older adults, by extension, reducing the risk of associated disabilities [42,44]. Additionally, a longitudinal study conducted in the United States has indicated that high hPDI is associated with the effective regulation of oxidative stress and systemic inflammation, thereby potentially lowering the risk of chronic obstructive pulmonary disease (COPD) [45]. This association may be linked to the reduced consumption of substances such as saturated fats, sodium, and nitrites. The aforementioned studies substantiate our findings. Nevertheless, certain scholars, including Van and Samson et al., contend that plant-based dietary patterns, lacking essential nutrients like vitamin B12, EPA, and dietary calcium, might negate some of the benefits of plant-based diets [46,47,48,49,50]. Consequently, it remains to be determined whether a high-hPDI diet can reduce the risk of ADL disability in older adults by preventing and alleviating chronic degenerative diseases, warranting further investigation in future research.

Simultaneously, our findings indicate that a high uPDI is correlated with an increased risk of ADL disability among older adults. Existing research supports our findings [40,51]. In comparison to high-quality plant-based dietary patterns rich in fruits, vegetables, and whole grains that are linked to health benefits, lower-quality plant-based diets, comprising refined grains, sugar-sweetened beverages, and snacks, correlate with a higher risk of chronic degenerative diseases. This elevated risk, in turn, is likely to result in a greater propensity for disability among older adults. The glycemic index (GI) is employed to assess the rate of carbohydrate digestion and subsequent absorption into the bloodstream [52]. Glycemic load (GL) is the product of the GI and the total available carbohydrate content in a specified food portion. It measures the degree and duration of blood glucose elevation following the consumption of a specific amount of carbohydrate-rich food [52,53]. A substantial body of research indicates that the consumption of foods with elevated glycemic index (GI) and glycemic load (GL) is significantly linked to the risk of obesity, metabolic syndrome, cardiovascular diseases, low-grade systemic inflammation, and cognitive impairments [54,55,56,57].

After stratifying participants by age, our analyses revealed that the association between hPDI and the risk of ADL disability in older adults was statistically significant in all age groups. hPDI exhibited a more pronounced protective effect in mitigating the risk of ADL disability among individuals below the age of 80, relative to those aged 80 and above. Moreover, high uPDI was only found to exert a significant adverse impact on the risk of ADL disability exclusively in older adults aged less than 80 years. We believe this may be due to two reasons. Firstly, the influence of chronic degenerative diseases tends to decrease with advancing age in the elderly population. Chronic degenerative diseases (e.g., cardiovascular disease, obesity, diabetes) are significant risk factors for ADL disability in older adults, and a high uPDI is associated with an increased risk of these diseases. However, a longitudinal Chinese study indicates that older elderly people are less prone to ADL disability attributed to chronic degenerative diseases compared to younger elderly people [58]. This may be because social determinants are likely to exert a more significant influence on the progression of ADL disability in older elderly people with advancing age [59,60]. For example, older elderly people typically possess broader social networks and are more likely to garner social support, facilitating their access to medical assistance and reducing the harm of chronic degenerative diseases. Secondly, it is influenced by the quantity of food consumed by older adults. Anorexia nervosa, characterized by a diminished appetite and reduced food intake in older adults, exhibits an increased incidence with advancing age, disproportionately affecting the oldest old [61,62,63]. In this study, the FFQ was utilized to determine the uPDI, considering only the frequency of food consumption and not the quantity. Thus, the adverse impact of a high uPDI may be attenuated when assessing its influence on ADL disability among adults aged 80 and above.

## 5. Innovations and Limitations

The present study possesses several notable strengths. Firstly, our study used nationally representative data. Secondly, we employed a cohort study design, which offers a robust framework for causal inference. Lastly, to the best of our knowledge, this study pioneers the use of PDI, hPDI, and uPDI to investigate the direct link between plant-based dietary patterns and disability outcomes. However, this study also has some limitations. Firstly, the reliance on self-assessment questionnaires for data collection in this study may have introduced recall bias. Secondly, the PDI, hPDI, and uPDI in this study were calculated based on baseline dietary data only and did not take into account subsequent dietary changes in participants. Thirdly, although the study considered the type and quality of dietary data, it did not factor in the portion sizes, which could influence the overall nutritional intake. Lastly, the study did not consider the potential impact of dietary supplements on the outcomes, which may have introduced additional variability.

## 6. Conclusions

The findings of this study demonstrate that a high hPDI confers a protective effect against the risk of ADL disability among older adults, whereas a high uPDI is correlated with an increased risk for ADL disability in this population. In parallel, a high hPDI had a more pronounced effect in reducing the risk of ADL disability in the younger elderly population aged below 80, in comparison to those over 80. Moreover, uPDI was only found to negatively impact the risk of ADL disability in younger older adults aged less than 80 years. In conclusion, our study found that increasing the intake of a healthy plant-based diet while decreasing the intake of an animal-based diet has a protective effect on preventing and reducing ADL disability in older adults.

## Figures and Tables

**Table 1:** Baseline characteristics of participants by different PDI groups.

Characteristics	Total, n (%)	T1, n (%)	T2, n (%)	T3, n (%)	*χ* ^2^	*p*-Value
ADL, n (%)						
Non-disability	1435 (71.6)	440 (68.4)	438 (72.0)	557 (74.0)	5.32	0.070
Disability	569 (28.4)	203 (31.6)	170 (28.0)	196 (26.0)		
Sex, n (%)						
Male	949 (47.4)	295 (45.9)	269 (44.2)	385 (51.1)	7.23	0.027
Female	1055 (52.6)	348 (54.1)	339 (55.8)	368 (48.9)		
Age, n (%)						
<80 years	1438 (71.8)	416 (64.7)	430 (70.7)	592 (78.6)	33.63	<0.001
≥80 years	566 (28.2)	227 (35.3)	178 (29.3)	161 (21.4)		
Residence, n (%)						
Urban	243 (12.1)	62 (9.6)	91 (15.0)	90 (12.0)	19.92	<0.001
Town	411 (20.5)	108 (16.8)	128 (21.1)	175 (23.2)		
Rural	1350 (67.4)	473 (73.6)	389 (64.0)	488 (64.8)		
Financial status, n (%)						
Sufficient	1564 (78.0)	473 (73.6)	479 (78.8)	612 (81.3)	12.32	0.002
Insufficient	440 (22.0)	170 (26.4)	129 (21.2)	141 (18.7)		
Co-residence, n (%)						
With household Member(s)	1673 (83.5)	520 (80.9)	502 (82.6)	651 (86.5)	9.29	0.054
Solitary	317 (15.8)	118 (18.4)	100 (16.4)	99 (13.1)		
In an institution	14 (0.7)	5 (0.8)	6 (1.0)	3 (0.4)		
Marital status, n (%)						
Married and living with a spouse	1162 (58.0)	329 (51.2)	347 (57.1)	486 (64.5)	40.82	<0.001
Separated	61 (3.0)	34 (5.3)	12 (2.0)	15 (2.0)		
Divorced	6 (0.3)	3 (0.5)	3 (0.5)	0 (0.0)		
Widowed	755 (37.7)	271 (42.1)	237 (39.0)	247 (32.8)		
Never married	20 (1.0)	6 (0.9)	9 (1.5)	5 (0.7)		
Currently smoking, n (%)						
Yes	454 (22.7)	129 (20.1)	128 (21.1)	197 (26.2)	8.64	0.013
No	1550 (77.3)	514 (79.9)	480 (78.9)	556 (73.8)		
Currently drinking, n (%)						
Yes	455 (22.2)	135 (21.0)	140 (23.0)	170 (22.6)	0.84	0.656
No	1559 (77.8)	508 (79.0)	468 (77.0)	583 (77.4)		
Physical exercise, n (%)						
Yes	705 (35.2)	190 (29.5)	242 (39.8)	273 (36.3)	15.02	<0.001
No	1299 (64.8)	453 (70.5)	366 (60.2)	480 (63.7)		
Body mass index, n (%)						
Underweight	433 (21.6)	201 (31.3)	116 (19.1)	116 (15.4)	72.31	<0.001
Normal	1142 (57.0)	354 (55.1)	342 (56.3)	446 (59.2)		
Overweight	338 (16.9)	72 (11.2)	121 (19.9)	145 (19.3)		
Obese	91 (4.5)	16 (2.5)	29 (4.8)	46 (6.1)		

**Table 2:** Association of baseline PDI, hPDI, and uPDI with incidence of ADL disability risk.

		T1	T2		T3	
			HR (95%CI)	*p*-Value	HR (95%CI)	*p*-Value
PDI						
	Model 1	1	0.89 (0.73–1.09)	0.27	0.81 (0.67–0.98)	0.03
	Model 2	1	0.87 (0.70–1.07)	0.17	0.88 (0.71–1.07)	0.20
hPDI						
	Model 1	1	0.82 (0.68–0.99)	0.04	0.53 (0.43–0.66)	<0.01
	Model 2	1	0.86 (0.71–1.04)	0.11	0.61 (0.49–0.75)	<0.01
uPDI						
	Model 1	1	0.99 (0.80–1.23)	0.95	1.31 (1.08–1.60)	0.01
	Model 2	1	0.98 (0.79–1.23)	0.89	1.33 (1.07–1.64)	0.01

Note: Participants were then categorized into three groups (T1, T2, and T3) based on their scores. T1 refers to the lowest scoring of the three groups; T2 refers to the second highest scoring of the three groups; T3 refers to the highest scoring of the three groups. Model 1 is not adjusted for covariates. Model 2 adjusts for sex, age, residence, financial status, co-residence, marital status, smoking, drinking, physical exercise, and body mass index (BMI).

## Data Availability

All data used in this study are publicly available on the CLHLS website https://opendata.pku.edu.cn/dataverse/CHADS (accessed on 1 January 2024).

## Supplementary Materials

The following supporting information can be downloaded at: https://www.mdpi.com/article/10.3390/nu16234011/s1, Table S1: Simplified Food Frequency questionnaire and Scoring of plant-based diet indices; Table S2: Baseline characteristics of participants by different hPDI groups; Table S3: Baseline characteristics of participants by different uPDI groups; Figure S1: Kaplan-Meier survival curves for the PDI group; Figure S2: Kaplan-Meier survival curves for the hPDI group; Figure S3: Kaplan-Meier survival curves for the hPDI group.

## Abbreviations

ADL	Activities of Daily Living
PDI	Plant-Based Diet Index
hPDI	healthy Plant-Based Diet Index
uPDI	unhealth Plant-Based Diet Index
HR	Hazard Ratio
CI	Confidence Interval

## References

[1] World Health Organization (2001). International Classification of Functioning, Disability and Health (ICF).

[2] Zhang Y., Xiong Y., Yu Q., Shen S., Chen L., Lei X. (2021). The activity of daily living (ADL) subgroups and health impairment among Chinese elderly: A latent profile analysis. BMC Geriatr..

[3] United Nations (2015). World Population Ageing 2015.

[4] Qian J.H., Wu K., Luo H.Q., Cao P.Y., Ren X.H. (2016). Prevalence of loss of activities of daily living and influencing factors in elderly population in China. Zhonghua Liu Xing Bing Xue Za Zhi.

[5] Tian G., Li R., Cui Y., Zhou T., Shi Y., Yang W., Ma Y., Shuai J., Yan Y. (2022). Association between disability, social support and depressive symptoms in Chinese older adults: A national study. Front. Public Health.

[6] Gao Y., Du L., Cai J., Hu T. (2022). Effects of functional limitations and activities of daily living on the mortality of the older people: A cohort study in China. Front. Public Health.

[7] Lisko I., Kulmala J., Annetorp M., Ngandu T., Mangialasche F., Kivipelto M. (2021). How can dementia and disability be prevented in older adults: Where are we today and where are we going?. J. Intern. Med..

[8] Carmona-Torres J.M., Rodríguez-Borrego M.A., Laredo-Aguilera J.A., López-Soto P.J., Santacruz-Salas E., Cobo-Cuenca A.I. (2019). Disability for basic and instrumental activities of daily living in older individuals. PLoS ONE.

[9] Agarwal P., Wang Y., Buchman A.S., Bennett D.A., Morris M.C. (2019). Dietary Patterns and Self-Reported Incident Disability in Older Adults. J. Gerontol. Ser. A Biol..

[10] Loyd C., Markland A.D., Zhang Y., Fowler M., Harper S., Wright N.C., Carter C.S., Buford T.W., Smith C.H., Kennedy R. (2020). Prevalence of Hospital-Associated Disability in Older Adults: A Meta-analysis. J. Am. Med. Dir. Assoc..

[11] Ren L., Tang Y., Yang R., Hu Y., Wang J., Li S., Yu M., Jiang Y., Liu Z., Wu Y. (2023). Plant-based dietary pattern and low muscle mass: A nation-wide cohort analysis of Chinese older adults. BMC Geriatr..

[12] Locke A., Schneiderhan J., Zick S.M. (2018). Diets for Health: Goals and Guidelines. Am. Fam. Physician.

[13] Féart C., Pérès K., Samieri C., Letenneur L., Dartigues J.F., Barberger-Gateau P. (2011). Adherence to a Mediterranean diet and onset of disability in older persons. Eur. J. Epidemiol..

[14] Tomata Y., Watanabe T., Sugawara Y., Chou W.T., Kakizaki M., Tsuji I. (2014). Dietary patterns and incident functional disability in elderly Japanese: The Ohsaki Cohort 2006 study. J. Gerontol. Ser. A Biol. Sci. Med. Sci..

[15] Milaneschi Y., Bandinelli S., Corsi A.M., Lauretani F., Paolisso G., Dominguez L.J., Semba R.D., Tanaka T., Abbatecola A.M., Talegawkar S.A. (2011). Mediterranean diet and mobility decline in older persons. Exp. Gerontol..

[16] Kondo K., Miura K., Okamura T., Okayama A., Ueshima H. (2023). Dietary Factors, Dietary Patterns, and Cardiovascular Disease Risk in Representative Japanese Cohorts: NIPPON DATA80/90. J. Atheroscler. Thromb..

[17] Okubo H., Inagaki H., Gondo Y., Kamide K., Ikebe K., Masui Y., Arai Y., Ishizaki T., Sasaki S., Nakagawa T. (2017). Association between dietary patterns and cognitive function among 70-year-old Japanese elderly: A cross-sectional analysis of the SONIC study. Nutr. J..

[18] Ozawa M., Ninomiya T., Ohara T., Doi Y., Uchida K., Shirota T., Yonemoto K., Kitazono T., Kiyohara Y. (2013). Dietary patterns and risk of dementia in an elderly Japanese population: The Hisayama Study. Am. J. Clin. Nutr..

[19] Hemler E.C., Hu F.B. (2019). Plant-Based Diets for Cardiovascular Disease Prevention: All Plant Foods Are Not Created Equal. Curr. Atheroscler. Rep..

[20] Satija A., Bhupathiraju S.N., Spiegelman D., Chiuve S.E., Manson J.E., Willett W., Rexrode K.M., Rimm E.B., Hu F.B. (2017). Healthful and Unhealthful Plant-Based Diets and the Risk of Coronary Heart Disease in U.S. Adults. J. Am. Coll. Cardiol..

[21] Lv Y., Rong S., Deng Y., Bao W., Xia Y., Chen L. (2023). Plant-based diets, genetic predisposition and risk of non-alcoholic fatty liver disease. BMC Med..

[22] Chinese Longitudinal Healthy Longevity Survey (CLHLS) Community Datasets (1998–2018). https://opendata.pku.edu.cn/dataset.xhtml?persistentId=doi:10.18170/DVN/WBO7LK&version=2.0.

[23] Zhao L., Wang J., Deng H., Chen J., Ding D. (2022). Depressive Symptoms and ADL/IADL Disabilities Among Older Adults from Low-Income Families in Dalian, Liaoning. Clin. Interv. Aging.

[24] Ćwirlej-Sozańska A., Wiśniowska-Szurlej A., Wilmowska-Pietruszyńska A., Sozański B. (2019). Determinants of ADL and IADL disability in older adults in southeastern Poland. BMC Geriatr..

[25] Thompson A.S., Tresserra-Rimbau A., Karavasiloglou N., Jennings A., Cantwell M., Hill C., Perez-Cornago A., Bondonno N.P., Murphy N., Rohrmann S. (2023). Association of Healthful Plant-Based Diet Adherence with Risk of Mortality and Major Chronic Diseases Among Adults in the UK. JAMA Netw. Open.

[26] Satija A., Hu F.B. (2018). Plant-based diets and cardiovascular health. Trends Cardiovasc. Med..

[27] Satija A., Bhupathiraju S.N., Rimm E.B., Spiegelman D., Chiuve S.E., Borgi L., Willett W.C., Manson J.E., Sun Q., Hu F.B. (2016). Plant-Based Dietary Patterns and Incidence of Type 2 Diabetes in US Men and Women: Results from Three Prospective Cohort Studies. PLoS Med..

[28] Wang F., Baden M.Y., Guasch-Ferré M., Wittenbecher C., Li J., Li Y., Wan Y., Bhupathiraju S.N., Tobias D.K., Clish C.B. (2022). Plasma metabolite profiles related to plant-based diets and the risk of type 2 diabetes. Diabetologia.

[29] Yang F., Jin J., Liu J., Lu X., Jiang H., Tan H., Zhou F., Zeng P. (2024). Plant-based index linked to fall risk in older Chinese adults: Cross-sectional evidence from a national cohort. Aging Clin. Exp. Res..

[30] Baden M.Y., Liu G., Satija A., Li Y., Sun Q., Fung T.T., Rimm E.B., Willett W.C., Hu F.B., Bhupathiraju S.N. (2019). Changes in Plant-Based Diet Quality and Total and Cause-Specific Mortality. Circulation.

[31] Cavanaugh J.E., Neath A.A., Lovric M. (2011). Akaike’s Information Criterion: Background, Derivation, Properties, and Refinements. International Encyclopedia of Statistical Science.

[32] Di Renzo L., Gualtieri P., De Lorenzo A. (2021). Diet, Nutrition and Chronic Degenerative Diseases. Nutrients.

[33] Gazerani P. (2020). Migraine and Diet. Nutrients.

[34] Adeloye D., Song P., Zhu Y., Campbell H., Sheikh A., Rudan I. (2022). Global, regional, and national prevalence of, and risk factors for, chronic obstructive pulmonary disease (COPD) in 2019: A systematic review and modelling analysis. Lancet. Respir. Med..

[35] Wang Y.B., Page A.J., Gill T.K., Melaku Y.A. (2023). The association between diet quality, plant-based diets, systemic inflammation, and mortality risk: Findings from NHANES. Eur. J. Nutr..

[36] Rigi S., Mousavi S.M., Benisi-Kohansal S., Azadbakht L., Esmaillzadeh A. (2021). The association between plant-based dietary patterns and risk of breast cancer: A case-control study. Sci. Rep..

[37] Di Renzo L., Gualtieri P., Romano L., Marrone G., Noce A., Pujia A., Perrone M.A., Aiello V., Colica C., De Lorenzo A. (2019). Role of Personalized Nutrition in Chronic-Degenerative Diseases. Nutrients.

[38] Shan Z., Li Y., Baden M.Y., Bhupathiraju S.N., Wang D.D., Sun Q., Rexrode K.M., Rimm E.B., Qi L., Willett W.C. (2020). Association Between Healthy Eating Patterns and Risk of Cardiovascular Disease. JAMA Intern. Med..

[39] Heianza Y., Zhou T., Sun D., Hu F.B., Qi L. (2021). Healthful plant-based dietary patterns, genetic risk of obesity, and cardiovascular risk in the UK biobank study. Clin. Nutr..

[40] Baden M.Y., Shan Z., Wang F., Li Y., Manson J.E., Rimm E.B., Willett W.C., Hu F.B., Rexrode K.M. (2021). Quality of Plant-Based Diet and Risk of Total, Ischemic, and Hemorrhagic Stroke. Neurology.

[41] Wang F., Zheng J., Yang B., Jiang J., Fu Y., Li D. (2015). Effects of Vegetarian Diets on Blood Lipids: A Systematic Review and Meta-Analysis of Randomized Controlled Trials. J. Am. Heart Assoc..

[42] Zhu A., Chen H., Shen J., Wang X., Li Z., Zhao A., Shi X., Yan L., Zeng Y., Yuan C. (2022). Interaction between plant-based dietary pattern and air pollution on cognitive function: A prospective cohort analysis of Chinese older adults. Lancet Reg. Health West. Pac..

[43] Rajaram S., Jones J., Lee G.J. (2019). Plant-Based Dietary Patterns, Plant Foods, and Age-Related Cognitive Decline. Adv. Nutr..

[44] Liang F., Fu J., Turner-McGrievy G., Wang Y., Qiu N., Ding K., Zeng J., Moore J.B., Li R. (2022). Association of Body Mass Index and Plant-Based Diet with Cognitive Impairment among Older Chinese Adults: A Prospective, Nationwide Cohort Study. Nutrients.

[45] Varraso R., Dumas O., Tabung F.K., Boggs K.M., Fung T.T., Hu F., Giovannucci E., Speizer F.E., Willett W.C., Camargo C.A. (2023). Healthful and Unhealthful Plant-Based Diets and Chronic Obstructive Pulmonary Disease in U.S. Adults: Prospective Study. Nutrients.

[46] Van Soest A.P.M., van de Rest O., Witkamp R.F., van der Velde N., de Groot L. (2023). The association between adherence to a plant-based diet and cognitive ageing. Eur. J. Nutr..

[47] Key T.J., Papier K., Tong T.Y.N. (2022). Plant-based diets and long-term health: Findings from the EPIC-Oxford study. Proc. Nutr. Soc..

[48] Sotos-Prieto M., Rodriguez-Artalejo F., Fung T.T., Meyer H.E., Hu F.B., Willett W.C., Bhupathiraju S.N. (2024). Plant-Based Diets and Risk of Hip Fracture in Postmenopausal Women. JAMA Netw. Open.

[49] Tong T.Y.N., Appleby P.N., Armstrong M.E.G., Fensom G.K., Knuppel A., Papier K., Perez-Cornago A., Travis R.C., Key T.J. (2020). Vegetarian and vegan diets and risks of total and site-specific fractures: Results from the prospective EPIC-Oxford study. BMC Med..

[50] Samson M.E., Yeung L.F., Rose C.E., Qi Y.P., Taylor C.A., Crider K.S. (2022). Vitamin B-12 malabsorption and renal function are critical considerations in studies of folate and vitamin B-12 interactions in cognitive performance: NHANES 2011–2014. Am. J. Clin. Nutr..

[51] Rosenfeld R.M., Juszczak H.M., Wong M.A. (2023). Scoping review of the association of plant-based diet quality with health outcomes. Front. Nutr..

[52] Augustin L.S.A., Kendall C.W.C., Jenkins D.J.A., Willett W.C., Astrup A., Barclay A.W., Björck I., Brand-Miller J.C., Brighenti F., Buyken A.E. (2015). Glycemic index, glycemic load and glycemic response: An International Scientific Consensus Summit from the International Carbohydrate Quality Consortium (ICQC). Nutr. Metab. Cardiovasc. Dis..

[53] Vlachos D., Malisova S., Lindberg F.A., Karaniki G. (2020). Glycemic Index (GI) or Glycemic Load (GL) and Dietary Interventions for Optimizing Postprandial Hyperglycemia in Patients with T2 Diabetes: A Review. Nutrients.

[54] Nikparast A., Etesami E., Rahmani J., Rafiei N., Ghanavati M. (2023). The association between plant-based diet indices and metabolic syndrome: A systematic review and dose-response meta-analysis. Front. Nutr..

[55] Salehin S., Rasmussen P., Mai S., Mushtaq M., Agarwal M., Hasan S.M., Salehin S., Raja M., Gilani S., Khalife W.I. (2023). Plant Based Diet and Its Effect on Cardiovascular Disease. Int. J. Environ. Res. Public Health.

[56] Bouvard V., Loomis D., Guyton K.Z., Grosse Y., Ghissassi F.E., Benbrahim-Tallaa L., Guha N., Mattock H., Straif K. (2015). Carcinogenicity of consumption of red and processed meat. Lancet. Oncol..

[57] Gaylor C.M., Benton D., Brennan A., Young H.A. (2022). The impact of glycaemic load on cognitive performance: A meta-analysis and guiding principles for future research. Neurosci. Biobehav. Rev..

[58] Pan C., Cao N., Kelifa M.O., Luo S. (2023). Age and cohort trends in disability among Chinese older adults. Front. Public Health.

[59] Chen Y., Sloan F.A. (2015). Explaining Disability Trends in the U.S. Elderly and Near-Elderly Population. Health Serv. Res..

[60] Martin L.G., Freedman V.A., Schoeni R.F., Andreski P.M. (2010). Trends in disability and related chronic conditions among people ages fifty to sixty-four. Health Aff..

[61] Dent E., Hoogendijk E.O., Visvanathan R., Wright O.R.L. (2019). Malnutrition Screening and Assessment in Hospitalised Older People: A Review. J. Nutr. Health Aging.

[62] Fielding R.A., Landi F., Smoyer K.E., Tarasenko L., Groarke J. (2023). Association of anorexia/appetite loss with malnutrition and mortality in older populations: A systematic literature review. J. Cachexia Sarcopenia Muscle.

[63] Wysokiński A., Sobów T., Kłoszewska I., Kostka T. (2015). Mechanisms of the anorexia of aging-a review. Age.

