# Supplementary material for: Association of Plant-Based Dietary Patterns with Activities of Daily Living Disability in Older Adults Based on a 10-Year Cohort Study
Source: Nutrients. 2024 Nov 23;16(23):4011. doi: 10.3390/nu16234011 (PMC11643895; doi:10.3390/nu16234011)
Supplement: Supplementary file 1 [file nutrients-16-04011-s001.zip › nutrients-3311547-supplementary.pdf]

Contents

1. Table S1. Simplified Food Frequency questionnaire and Scoring of plant-based diet indices ·· 1

2. Table S2 Baseline characteristics of participants by different hPDI groups ··· 3

3. Table S3 Baseline characteristics of participants by different uPDI groups ··· 5

4. Figure S1 Kaplan-Meier survival curves for the PDI group ·········· 7

5. Figure S2 Kaplan-Meier survival curves for the hPDI group········· 8

6. Figure S3 Kaplan-Meier survival curves for the hPDI group········· 9

Table S1. Simplified Food Frequency questionnaire and Scoring of plant-based diet indices

Types	Food	Frequency	PDI	hPDI	uPDI
Healthful plant-based food	Whole grain	Yes	5	5	1
		No	1	1	5
	Vegetable oil	Yes	5	5	1
		No	1	1	5
	Fresh fruit	Almost everyday	5	5	1
		Except winter	4	4	2
		Occasionally	2	2	4
		Rarely or never	1	1	5
	Vegetable	Almost everyday	5	5	1
		Except winter	4	4	2
		Occasionally	2	2	4
		Rarely or never	1	1	5
	Legume	Almost everyday	5	5	1
		≥1 time/week	4	4	2
		≥1 time/month	3	3	3
		Occasionally	2	2	4
		Rarely or never	1	1	5
	Garlic	Almost everyday	5	5	1
		≥1 time/week	4	4	2
		≥1 time/month	3	3	3
		Occasionally	2	2	4
		Rarely or never	1	1	5
	Nut products	Almost everyday	5	5	1
		≥1 time/week	4	4	2
		≥1 time/month	3	3	3
		Occasionally	2	2	4
		Rarely or never	1	1	5
	Tea	Almost everyday	5	5	1
		≥1 time/week	4	4	2
		≥1 time/month	3	3	3
		Occasionally	2	2	4
		Rarely or never	1	1	5
Unhealthful plant-based	Refined grain	Yes	5	1	5
		No	1	5	1

food	Salt-preserved vegetable	Almost everyday	5	1	5
		≥1 time/week	4	2	4
		≥1 time/month	3	3	3
		Occasionally	2	4	2
		Rarely or never	1	5	1
	Sugar	Almost everyday	5	1	5
		≥1 time/week	4	2	4
		≥1 time/month	3	3	3
		Occasionally	2	4	2
		Rarely or never	1	5	1
Animal-based food	Animal fat	Yes	1		
		No	5		
	Meat	Almost everyday	1		
		≥1 time/week	2		
		≥1 time/month	3		
		Occasionally	4		
		Rarely or never	5		
	Fish	Almost everyday	1		
		≥1 time/week	2		
		≥1 time/month	3		
		Occasionally	4		
		Rarely or never	5		
	Egg	Almost everyday	1		
		≥1 time/week	2		
		≥1 time/month	3		
		Occasionally	4		
		Rarely or never	5		
	Milk products	Almost everyday	1		
		≥1 time/week	2		
		≥1 time/month	3		
		Occasionally	4		
		Rarely or never	5		

Table S2 Baseline characteristics of participants by different hPDI groups

Characteristic	Total, N(%)	T1,N(%)	T2, N(%)	T3, N(%)	$\chi 2$	*P*-Value
ADL, N(%)						
Non-disability	1435 (71.6)	397 (64.4)	499 (70.1)	539 (79.7)	38.30	<0.001
Disability	569 (28.4)	219 (35.6)	213 (29.9)	137 (20.3)		
Sex, N(%)						
Male	949 (47.4)	278 (45.1)	342 (48.0)	329 (48.7)	1.82	0.402
Female	1055 (52.6)	338 (54.9)	370 (52.0)	347 (51.3)		
Age, N(%)						
<80 years	1438 (71.8)	410 (66.6)	503 (70.6)	525 (77.7)	20.28	<0.001
≥80 years	566 (28.2)	206 (33.4)	209 (29.4)	151 (22.3)		
Residence, N(%)						
Urban	243 (12.1)	69 (11.2)	97 (13.6)	77 (11.4)		
Town	411 (20.5)	76 (12.3)	136 (19.1)	199 (29.4)	63.38	<0.001
Rural	1350 (67.4)	471 (76.5)	479 (67.3)	400 (59.2)		
Financial status, N(%)						
Sufficient	1564 (78.0)	479 (77.8)	557 (78.2)	528 (78.1)	0.05	0.978
Insufficient	440 (22.0)	137 (22.2)	155 (21.8)	148 (21.9)		
Co-residence, N(%)						
With household Member(s)	1673 (83.5)	506 (82.1)	591 (83.0)	576 (85.2)		
Solitary	317 (15.8)	110 (17.9)	111 (15.6)	96 (14.2)	12.65	0.013
In an institution	14 (0.7)	0 (0.0)	10 (1.4)	4 (0.6)		
Marital status, N(%)						

Married and living with a spouse	1162 (58.0)	333 (54.1)	413 (58.0)	416 (61.5)		
Separated	61 (3.0)	15 (2.4)	21 (2.9)	25 (3.7)		
Divorced	6 (0.3)	5 (0.8)	0 (0.0)	1 (0.1)	19.48	0.012
Widowed	755 (37.7)	256 (41.6)	269 (37.8)	230 (34.0)		
Never married	20 (1.0)	7 (1.1)	9 (1.3)	4(0.6)		
Currently smoking, N(%)						
Yes	454 (22.7)	125 (20.3)	171 (24.0)	158 (23.4)	2.92	0.233
No	1550 (77.3)	491 (79.7)	541 (76.0)	518 (76.6)		
Currently drinking, N(%)						
Yes	455 (22.2)	126 (20.5)	177 (24.9)	142 (21.0)	4.56	0.102
No	1559 (77.8)	490 (79.5)	535 (75.1)	534 (79.0)		
Physical exercise, N(%)						
Yes	705 (35.2)	200 (32.5)	260 (36.5)	245 (36.2)	2.88	0.237
No	1299 (64.8)	416 (67.5)	452 (63.5)	431 (63.8)		
Body mass index, N(%)						
Underweight	433 (21.6)	157 (25.5)	149 (20.9)	127 (18.8)		
Normal	1142 (57.0)	320 (51.9)	413 (58.0)	409 (60.5)	14.17	0.028
Overweight	338 (16.9)	105 (17.0)	117 (16.4)	116 (17.2)		
Obese	91 (4.5)	34 (5.5)	33 (4.6)	24 (3.6)		

Table S3 Baseline characteristics of participants by different uPDI groups

Characteristic	Total, N(%)	T1, N(%)	T2, N(%)	T3, N(%)	$\chi 2$	*P*-Value
ADL, N(%)						
Non-disability	1435 (71.6)	461 (74.1)	476 (74.3)	498 (67.2)	11.20	0.004
Disability	569 (28.4)	161 (25.9)	165 (25.7)	243 (32.8)		
Sex, N(%)						
Male	949 (47.4)	318 (51.1)	300 (46.8)	331 (44.7)	5.77	0.056
Female	1055 (52.6)	304 (48.9)	341 (53.2)	410 (55.3)		
Age, N(%)						
<80 years	1438 (71.8)	468 (75.2)	448 (69.9)	522 (70.4)	5.46	0.065
≥80 years	566 (28.2)	154 (24.8)	193 (30.1)	219 (29.6)		
Residence, N(%)						
Urban	243 (12.1)	133 (21.4)	58 (9.0)	52 (7.0)		
Town	411 (20.5)	159 (25.6)	141 (22.0)	111 (15.0)	115.99	<0.001
Rural	1350 (67.4)	330 (53.1)	442 (69.0)	578 (78.0)		
Financial status, N(%)						
Sufficient	1564 (78.0)	544 (87.5)	490 (76.4)	530 (71.5)	51.52	<0.001
Insufficient	440 (22.0)	78 (12.5)	151 (23.6)	211 (28.5)		
Co-residence, N(%)						
With household Member(s)	1673 (83.5)	542 (87.1)	539 (84.1)	592 (79.9)		
Solitary	317 (15.8)	73 (11.7)	98 (15.3)	146 (19.7)	18.49	<0.001
In an institution	14 (0.7)	7 (1.1)	4 (0.6)	3 (0.4)		
Marital status, N(%)						

Married and living with a spouse	1162 (58.0)	390 (62.7)	375 (58.5)	390 (62.7)		
Separated	61 (3.0)	25 (4.0)	19 (3.0)	25 (4.0)		
Divorced	6 (0.3)	1 (0.2)	3 (0.5)	1 (0.2)	20.09	0.010
Widowed	755 (37.7)	202 (32.5)	238 (37.1)	202 (32.5)		
Never married	20 (1.0)	4 (0.6)	6 (0.9)	4 (0.6)		
Currently smoking, N(%)						
Yes	454 (22.7)	141 (22.7)	141 (22.0)	172 (23.2)	0.29	0.865
No	1550 (77.3)	481 (77.3)	500 (78.0)	569 (76.8)		
Currently drinking, N(%)						
Yes	455 (22.2)	148 (23.8)	154 (24.0)	143 (19.3)	5.76	0.056
No	1559 (77.8)	474 (76.2)	487 (76.0)	598 (80.7)		
Physical exercise, N(%)						
Yes	705 (35.2)	283 (45.5)	195 (30.4)	227 (30.6)	42.12	<0.001
No	1299 (64.8)	339 (54.5)	446 (69.6)	514 (69.4)		
Body mass index, N(%)						
Underweight	433 (21.6)	104 (16.7)	137 (21.4)	192 (25.9)		
Normal	1142 (57.0)	379 (60.9)	364 (56.8)	399 (53.8)	18.04	0.006
Overweight	338 (16.9)	113 (18.2)	107 (16.7)	118 (15.9)		
Obese	91 (4.5)	26 (4.2)	33 (5.1)	32 (4.3)		

Figure S1 Kaplan-Meier survival curves for the PDI group

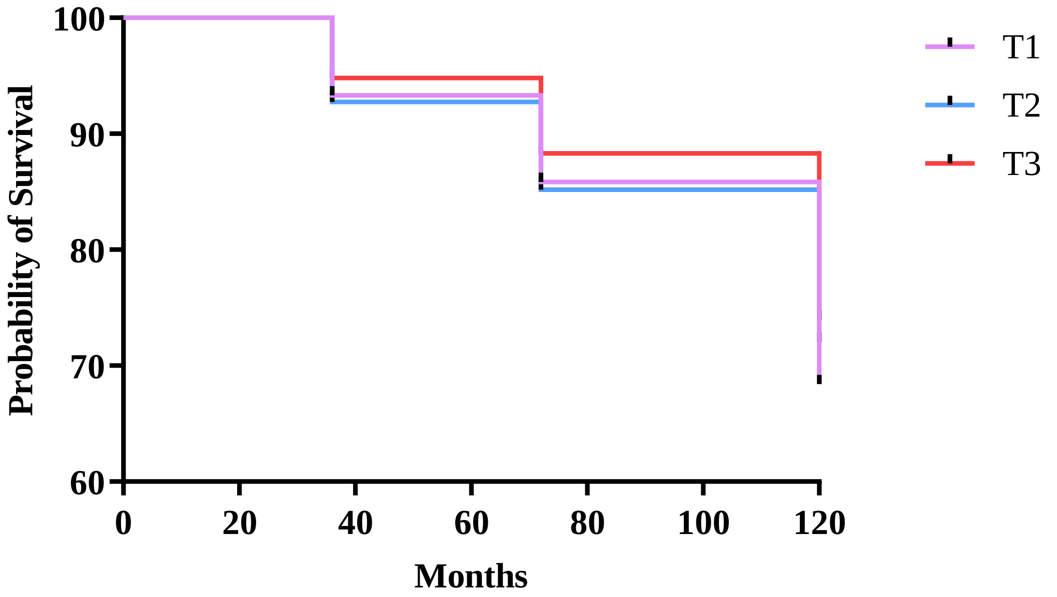

In this study, PDI was further stratified into three groups (T1, T2, and T3) based on the tertiles of the subjects' scores. T1 is PDI ≤ 48; T2 is PDI > 48. but ≤ 53; T3 is PDI > 53.

Figure S2 Kaplan-Meier survival curves for the hPDI group

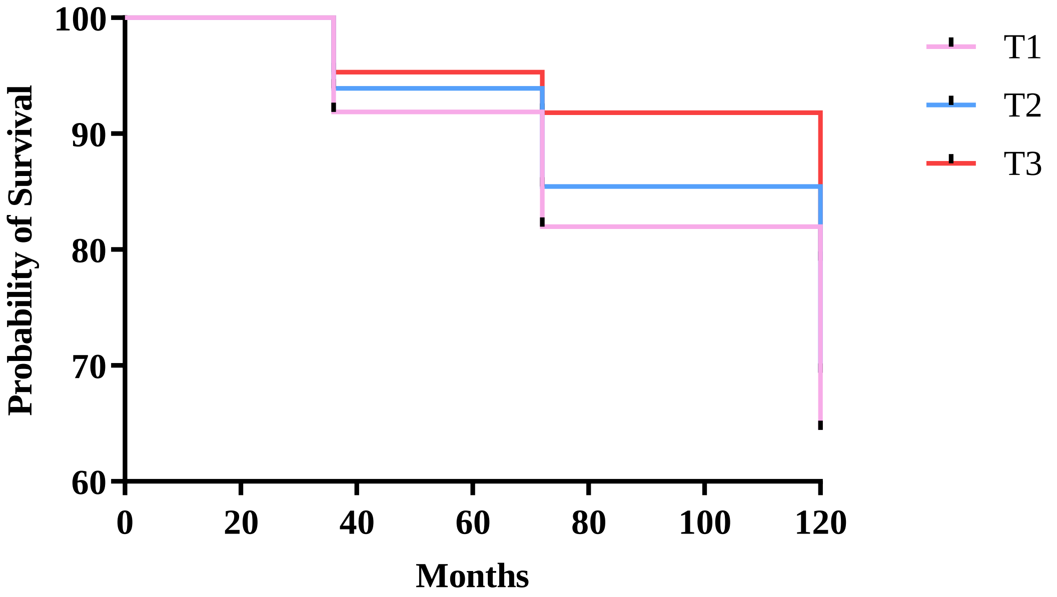

In this study, hPDI was further stratified into three groups (T1, T2, and T3) based on the tertiles of the subjects' scores. T1 is hPDI ≤ 50; T2 is hPDI > 53. but ≤ 56; T3 is hPDI > 56.

Figure S3 Kaplan-Meier survival curves for the uPDI group

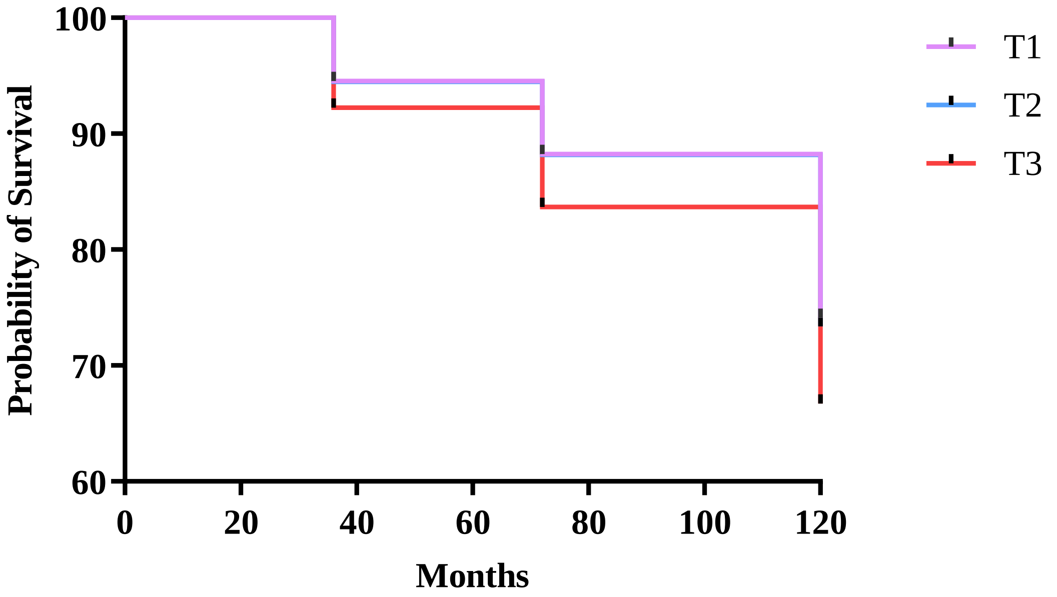

In this study, hPDI was further stratified into three groups (T1, T2, and T3) based on the tertiles of the subjects' scores. T1 is uPDI ⩽ 42; T2 is uPDI > 42. but ⩽ 48; T3 is uPDI > 48.
